# Nuclear Localization of the Mitochondrial Factor HIGD1A during Metabolic Stress

**DOI:** 10.1371/journal.pone.0062758

**Published:** 2013-04-30

**Authors:** Kurosh Ameri, Anthony M. Rajah, Vien Nguyen, Timothy A. Sanders, Arman Jahangiri, Michael DeLay, Matthew Donne, Hwa J. Choi, Kathryn V. Tormos, Yerem Yeghiazarians, Stefanie S. Jeffrey, Paolo F. Rinaudo, David H. Rowitch, Manish Aghi, Emin Maltepe

**Affiliations:** 1 Department of Pediatrics/Neonatology, University of California San Francisco, San Francisco, California, United States of America; 2 Department of Neurological Surgery, University of California San Francisco, San Francisco, California, United States of America; 3 Department of Developmental and Stem Cell Biology, University of California San Francisco, San Francisco, California, United States of America; 4 Department of Medicine/Cardiology, University of California San Francisco, San Francisco, California, United States of America; 5 Department of Surgery, Stanford University School of Medicine, Palo Alto, California, United States of America; 6 Department of Obstetrics, Gynecology and Reproductive Sciences, University of California San Francisco, San Francisco, California, United States of America; UAE University, United Arab Emirates

## Abstract

Cellular stress responses are frequently governed by the subcellular localization of critical effector proteins. Apoptosis-inducing Factor (AIF) or Glyceraldehyde 3-Phosphate Dehydrogenase (GAPDH), for example, can translocate from mitochondria to the nucleus, where they modulate apoptotic death pathways. Hypoxia-inducible gene domain 1A (HIGD1A) is a mitochondrial protein regulated by Hypoxia-inducible Factor-1α (HIF1α). Here we show that while HIGD1A resides in mitochondria during physiological hypoxia, severe metabolic stress, such as glucose starvation coupled with hypoxia, in addition to DNA damage induced by etoposide, triggers its nuclear accumulation. We show that nuclear localization of HIGD1A overlaps with that of AIF, and is dependent on the presence of BAX and BAK. Furthermore, we show that AIF and HIGD1A physically interact. Additionally, we demonstrate that nuclear HIGD1A is a potential marker of metabolic stress *in vivo,* frequently observed in diverse pathological states such as myocardial infarction, hypoxic-ischemic encephalopathy (HIE), and different types of cancer. In summary, we demonstrate a novel nuclear localization of HIGD1A that is commonly observed in human disease processes *in vivo*.

## Introduction

Ischemic heart disease, stroke and cancer are associated with cellular hypoxia and nutrient/glucose deprivation [1], [2], [3], [4]. The Hypoxia Inducible Factor (HIF) family of transcriptional regulators modulates the survival of cells in response to these stressors [5] [6], [7], [8]. HIFs are heterodimers consisting of oxygen sensitive, labile α subunits complexed with stable β subunits. With increasing levels of oxygen, HIF-α subunits are hydroxylated at conserved proline residues, mediated by a family of prolyl-4-hydroxylase domain (PHD) enzymes. Hydroxylated HIFα is then recognized and targeted for proteasomal degradation by the von Hippel-Lindau protein (pVHL) complex. Under hypoxic conditions, PHD activity ceases and the rate of hydroxylation declines leading to HIF-α accumulation [9], [10], [11]. Once stabilized, HIF-1α heterodimerizes with HIF-1β, and regulates the expression of scores of adaptive/survival genes. Therapeutic manipulation of HIF-hydroxylases therefore has obvious appeal [12].

The maintenance of cellular bioenergetics within tissues and organs is dependent on the coordinated interplay between multiple competing factors. Variations in substrate delivery and cellular metabolic rates can produce wide ranges of tissue oxygenation even in adults during non-stressful steady states [13], [14]. Furthermore, all of mammalian development occurs in a physiological hypoxia that does not compromise normal growth, but that is still dependent on HIF [15], [16]. Thus, cells possess multiple compensatory mechanisms to preserve cellular bioenergetics across a wide range of oxygen and glucose concentrations, and hypoxia and/or glucose deprivation only become pathologic when these countermeasures are exhausted [17]. During anoxia or ischemia, conditions that limit mitochondrial ATP production, adaptive mechanisms fail and cells undergo an “adaptation-to-death switch” [2], [18], [19], frequently in advance of true bioenergetic collapse. Interestingly, some cancer cells can escape this switch due to malfunctioning death pathways that contribute to their malignant progression [20], [21], [22].

Following bioenergetic compromise, multiple forms of cell death such as programmed cell death (PCD), or apoptosis, as well as necrosis are observed. In general, PCD can be classified as caspase-dependent or –independent [23]. Subcellular relocalization of effector proteins frequently drives these processes. In the intrinsic form of PCD, for example, signals from mitochondria, such as cytochrome c, are liberated to induce downstream caspase activation and subsequent cell death [24]. The export of cytochrome c during apoptosis is regulated by mitochondrial outer membrane permeabilization. This is determined, in part, by the opposing actions of the BCL-2 family of mitochondrial outer membrane proteins [25]. For example, while BCL-X_L_ inhibits mitochondrial outer membrane permeabilization, BAX and BAK promote it [26]. Similarly in caspase-independent PCD, mitochondrial factors such as AIF are released in response to toxic stimuli and directly promote apoptotic cell death following nuclear translocation [27]. During this “adaptation-to-death switch,” therefore, several factors contribute to cell death pathways via mechanisms dependent on altered subcellular localization [28], [29], [30], [31], [32], [33], [34], [35], [36].

*Higd1a* is a HIF-1 target gene originally described in cultured human cervical epithelial cells [37], and shown to be induced in hypoxic neuron-enriched primary cultures [38] as well as by nickel in mouse embryo fibroblasts [39]. HIGD1A is a ∼10 kDa mitochondrial inner membrane protein with adaptive functions during glucose deprivation [40], and promotes normal mitochondrial function via modulation of the mitochondrial γ-secretase complex [41]. The survival effect of HIGD1A is dependent on the level of HIGD1A expression [42]. Anti-apoptotic effects of HIGD1A in RAW264.7 macrophages have been shown to be associated with inhibition of cytochrome C release and reduced caspase activation [43]. In the rat spinal cord, HIGD1A expression increases after birth and during the first days of postnatal life during CNS remodeling [44]. During this period, many populations of neurons are known to undergo cell death with the number of apoptotic cells peaking just after birth and falling sharply the week thereafter. This trend suggests both cell death and survival roles for HIGD1A, depending on developmental stage and cellular microenvironment.

The subcellular localization of HIGD1A during severe stress has not been addressed to date. In this paper, we have investigated the localization of HIGD1A in mouse embryonic fibroblasts (MEFs) during metabolic stress, including glucose starvation coupled with prolonged hypoxia, in addition to etoposide induced DNA damage. We also examined the subcellular localization of HIGD1A during pathological states *in vivo*, including in human neonatal brains following HIE and infarcted mouse hearts, as well as human tumor xenografts and glioblastoma biopsies from patients before and after treatment with the antiangiogenesis agent Bevacizumab/Avastin. While found in mitochondria under basal conditions, we found that HIGD1A was frequently localized to the nucleus during these metabolically stressful states. Interestingly, HIGD1A and AIF interacted, and their nuclear localization was dependent on BAX and BAK. In summary, we describe a novel subcelluar localization for HIGD1A in the nucleus during severe stress *in vitro* and in several pathologic conditions associated with severe hypoxia and ischemia *in vivo*.

## Results

### HIGD1A is Regulated by HIF1α and Localizes to the Nucleus during Severe Stress

**Figure 1:**
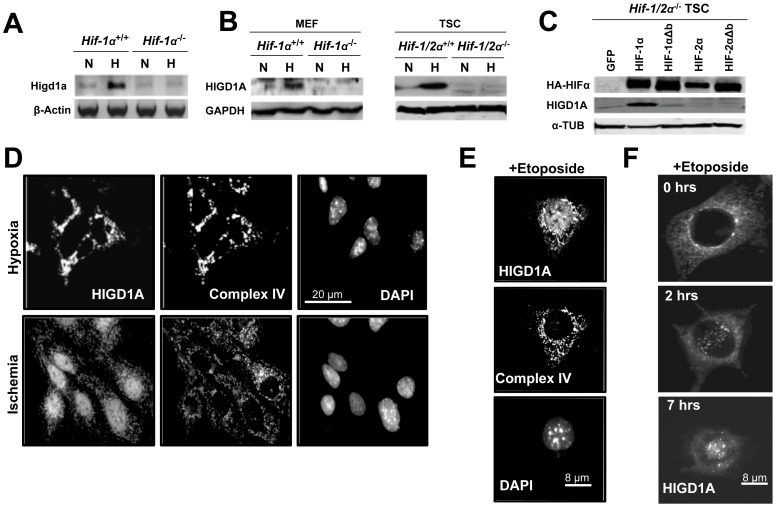
HIGD1A is a HIF-1 target localized to mitochondria under physiological conditions, but localizes to the nucleus during pathological stress in MEFs. (A) RT-PCR analysis of Higd1a mRNA expression in wild-type and *Hif-1α^−/−^* MEFs cultured under 20% O_2_ (N, normoxia) or 2% O_2_ (H, hypoxia). (B) Immunoblot analysis of HIGD1A protein expression in wild-type and *Hif-1α^−/−^* MEFs, as well as wild-type and *Hif-1/2α^−/−^* TSCs cultured under 20% O_2_ (N, normoxia) or 2% O_2_ (H, hypoxia). (C) Immunoblot analysis of HIGD1A protein expression in *Hif-1/2α^−/−^* TSCs stably expressing GFP, HIF-1α, HIF-2α or DNA-binding domain deficient versions of each (HIF-1αΔb and HIF-2αΔb). (D) Immunofluorescence microscopy of endogenous HIGD1A in control MEFs indicated a mitochondrial localization pattern during physiological hypoxia (2% O_2_), while more severe hypoxia (1% oxygen) coupled with glucose starvation (Ischemia) triggered its nuclear localization. Complex IV subunit 2 immunoreactivity was used as a marker of mitochondria. Nuclei are identified with DAPI staining. (E) Immunofluorescence microscopy of endogenous HIGD1A indicated that HIGD1A exhibited a nuclear localization pattern following exposure to the DNA damaging agent Etoposide. (F) Live cell immunofluorescence microscopy of HIGD1A-GFP fusion protein indicated that prior to Etoposide exposure, HIGD1A protein is extranuclear, with nuclear accumulation observed as early as 2 hours following drug exposure, and increasing throughout the duration of the experiment.

To determine whether HIGD1A expression and induction is regulated by HIF1α or HIF2α, we used HIF-deficient MEFs and trophoblast stem cells (TSCs). As indicated by RTPCR in Fig. 1A, in contrast to wt cells (HIF+/+), HIF-1α deficient MEFs (HIF−/−) failed to induce Higd1a mRNA in hypoxia (Fig. 1B). Similarly, HIGD1A protein was only induced in wt (+/+) cells (MEFs and TSCs) when subjected to hypoxia (1% O_2_), but not in HIF deficient MEFs (−/−) or HIF-1/2α deficient TSCs (−/−). To determine whether HIGD1A was regulated specifically by HIF1α or HIF2α, we overexpressed HA-tagged HIF1α and HIF2α in HIF deficient TSCs (Hif-1/2α^−/−^) as previously described [45]. GFP overexpression in the same plasmid backbone served as a control. As indicated in Fig. 1C, HIGD1A protein was induced only when HIF1α was overexpressed. This induction of HIGD1A by HIF1α was dependent on canonical hypoxia response element binding, since overexpression of HIF1α that lacked the DNA binding basic domain (HIF-1aΔβ) failed to induce HIGD1A. These results demonstrate that HIGD1A is exclusively regulated by HIF1α via canonical target gene expression.

Several factors such as AIF [46] or GAPDH [28], [31], [34] become nuclear when cells are subjected to severe stress, such as during ischemia or exposure to DNA damaging agents such as etoposide. As shown in Fig. 1D, during physiological hypoxia (2% O_2_), endogenous HIGD1A was primarily localized to mitochondria in MEFs, confirming previous results. When subjected to ischemia (1% oxygen coupled with glucose starvation), or, as shown in Fig. 1E, the DNA damaging agent etoposide, however, endogenous HIGD1A localized to the nucleus, whereas complex IV subunit 2 of the electron transport chain remained mitochondrial under all conditions. To confirm these observations made with endogenous HIGD1A, and to rule out non-specific staining artifacts, we also examined MEFs that stably overexpressed a HIGD1A-GFP fusion protein. As indicated by live-cell epifluorescence microscopy in Fig. 1F, control cells prior to etoposide treatment demonstrated mitochondrial/cytoplasmic HIGD1A-GFP fluorescence. However, as early as 2 hours following treatment with etoposide, nuclear entry of HIGD1A-GFP fusion protein could be demonstrated, which increased throughout the duration of the experiment.

### HIGD1A Interacts with AIF and its Nuclear Localization is Dependent on BAX and BAK

**Figure 2:**
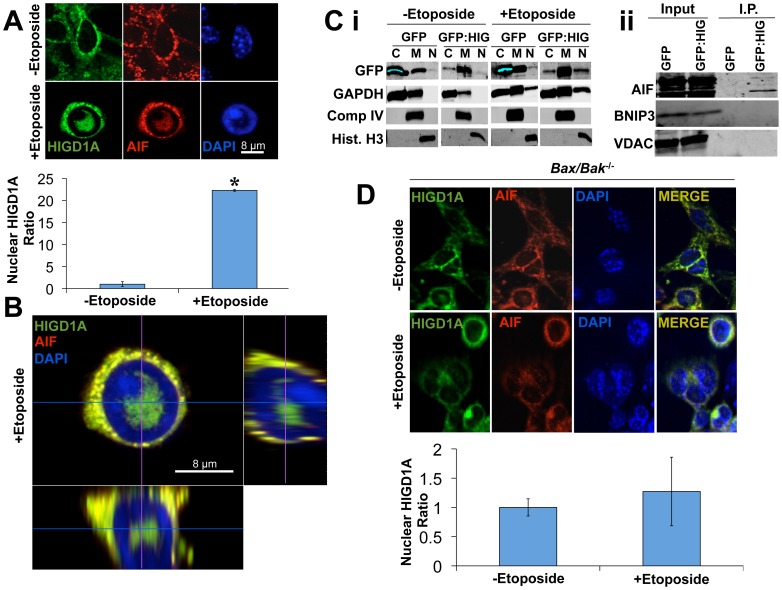
HIGD1A interacts with AIF and its nuclear localization is dependent on BAX and BAK. (A) Immunofluoresce confocal laser scanning microscopy of HIGD1A-GFP overexpressing MEFs indicated a co-localization of HIGD1A with AIF in mitochondria in the absence of Etoposide, with both proteins localizing to the nucleus following exposure to Etoposide (40 µM). Quantitation of the relative nuclear localization HIGD1A in the presence of etoposide versus control (- etoposide) in wild-type MEFs. * = p<0.05 (student’s t-test) (B) Confocal cross sections in *xy* (top left), *yz* (right), and *xz* (bottom) revealed co-localization of HIGD1A and AIF in nuclei of Etoposide exposed MEFs. (C i) Immunoblot analysis of fractionated cell extracts (C = cytoplasm, M = mitochondria, N = nucleus) obtained from MEFs overexpressing HIGD1A or GFP alone (as control) show that cells treated with Etoposide contain greater levels of HIGD1A-GFP fusion protein in the nucleus as compared to untreated control cells. GAPDH was expressed in cytoplasmic as well as mitochondrial fractions under control conditions, translocating to the nucleus following Etoposide exposure in both control and GFP:HIGD1A expressing MEFs. Histone H3 was used as a nuclear marker, Complex IV subunit II (Comp. IV) was used as a mitochondrial marker. (C ii) Immuno-precipitation assays with HIGD1A-GFP fusion protein or control GFP expressing MEFs with an anti-GFP antibody revealed specific interaction between HIGD1A and AIF *in vitro*. Two other mitochondrial factors, BNIP3 and VDAC, did not bind HIGD1A. (D) Immunofluorescence microscopy of *Bax/Bak^−/−^* MEFs revealed diminished nuclear localization of AIF and HIGD1A following exposure to Etoposide (40 µM) Quantitation of the relative nuclear localization HIGD1A in the presence of etoposide versus control (- etoposide) in *Bax/Bak* ^−/−^ MEFs.

To determine whether HIGD1A nuclear localization was associated with the nuclear translocation of AIF, we treated HIGD1A-GFP overexpressing cells with etoposide, and co-stained for GFP and AIF. As indicated in Fig. 2A, AIF and HIGD1A co-localized to mitochondria in untreated control cells. However, upon exposure to etoposide, co-staining for HIGD1A-GFP and AIF demonstrated the presence of both factors within the nucleus. Quantitation of nuclear HIGD1A relative to untreated control cells demonstrated significantly greater numbers of cells with nuclear HIGD1A when cells were treated with etoposide. Confocal immunofluorescence microscopy confirmed the nuclear col-localization of AIF and HIGD1A in response to Etoposide (Fig. 2B). We confirmed these observations of nuclear HIGD1A accumulation via biochemical fractionation followed by immunoblot analyses. As shown in Fig. 2Ci, in untreated control cells, HIGD1A-GFP fusion protein was localized primarily within mitochondrial fractions, although a small amount of cytoplasmic HIGD1A was also appreciated. Following etoposide treatment, however, a clear nuclear accumulation of HIGD1A was also appreciated, along with nuclear GAPDH (Fig. 2C). As subcellular markers, we used Histone H3 (H3), which is a nuclear protein, electron transport chain complex IV subunit 2, which is a mitochondrial protein, and GAPDH, which can localize to both the cytoplasm as well as mitochondria, and is known to translocate to the nucleus during severe stress [28], [31], [34]. As indicated in Fig. 2C, only HIGD1A and GAPDH became nuclear after etoposide treatment, whereas Histone H3 was solely present in the nucleus, and complex IV subunit 2 of the respiratory chain was only localized to mitochondria, irrespective of etoposide treatment. Together, these results confirm that HIGD1A is primarily a mitochondrial factor under basal conditions, but also accumulates in nuclei when cells experience severe stress.

Since AIF and HIGD1A were frequently observed within the same subcellular compartments under physiological and pathological conditions, we questioned whether HIGD1A and AIF might physically interact. As indicated in Fig. 2C ii, we were able to identify AIF as a HIGD1A-interacting protein following immunoprecipitation. Neither BNIP3, another mitochondrial HIF-1 target, nor VDAC, another mitochondrial outer membrane protein [47], interacted with HIGD1A, highlighting the specificity of the observed HIGD1A-AIF interaction.

Nuclear localization of AIF has been reported to be dependent on the presence of BAX and BAK [48]. We therefore interrogated BAX/BAK double knock out MEFs (*Bax/Bak^−/−^*) for nuclear localization of AIF and HIGD1A during etoposide-induced stress. As indicated in Fig. 2D, when *Bax/Bak^−/−^* cells were treated with etoposide, nuclear localization of AIF and HIGD1A was diminished when compared with wt MEFs. Quantitation of nuclear HIGD1A relative to untreated control cells demonstrated no significant differences between *Bax/Bak^−/−^* cells treated with etoposide and control cells. These results suggest that the nuclear localization of HIGD1A is dependent on BAX and BAK activity.

### HIGD1A Localizes to the Nucleus during Human Neonatal Hypoxic-ischemic Encephalopathy (HIE) *in vivo*

**Figure 3:**
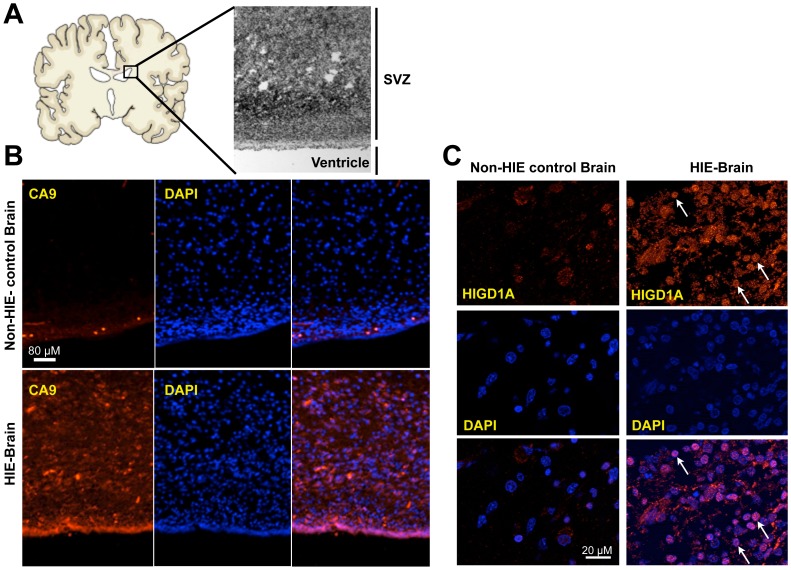
HIGD1A localizes to the nucleus in the setting of human neonatal hypoxic-ischemic encephalopathy (HIE) *in vivo*. (A) Schematic depiction of a coronal section through a human neonatal brain highlighting the subventricular zone (SVZ). (B) The SVZ of brains obtained from infants with HIE exhibited increased levels of the hypoxia marker CA9 compared with non-HIE control brains. (C)Immunofluorescence microscopy indicated low-level, extra-nuclear localization of endogenous HIGD1A in control human neonatal brains. Endogenous HIGD1A levels are increased in regions of human neonatal brains of infants who suffered HIE. Arrows indicate nuclear localization of endogenous HIGD1A in each. Experimental observations were made at least three times, and *in vivo* patient data are representative of three cases.

To investigate the relevance of nuclear HIGD1A localization *in vivo*, we examined tissue samples obtained from pathological conditions associated with hypoxia/ischemia, including HIE. Specifically, the sub-venticular zone (SVZ) of the brain was examined (Fig. 3A). As shown in Fig. 3B, the SVZ of human neonatal brains obtained from babies that succumbed to HIE were hypoxic as indicated by greater staining for carbonic anhydrase 9 (CA9)–a hypoxia marker regulated by HIF-1 [49]. As indicated in Fig. 3C, these regions demonstrated nuclear staining of HIGD1A, whereas control brains demonstrated weaker, non-nuclear HIGD1A staining.

### HIGD1A Localizes to the Nucleus during Murine Myocardial Infarction (MI) *in vivo*

**Figure 4:**
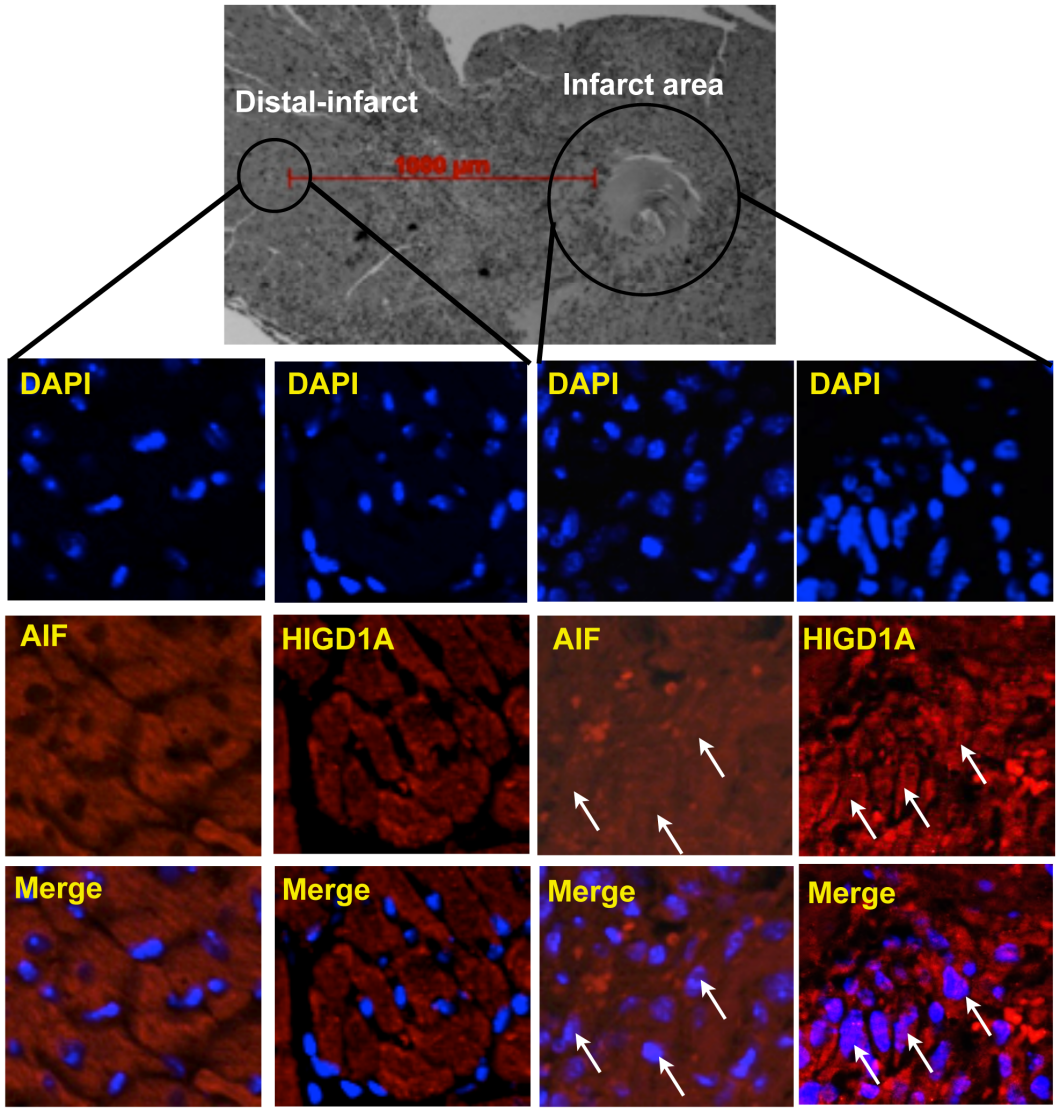
HIGD1A localizes to the nucleus in the setting of murine myocardial infarction (MI) *in vivo*. Top panel is a representative H&E stain of a mouse heart subjected to MI highlighting the area of infarct as well as regions distal to it where tissue was analyzed. As seen, in areas distal to the infarct, HIGD1A and AIF are expressed in a primarily extranuclear distribution. In the area of infarct, however, HIGD1A and AIF exhibit a much more diffuse localization that clearly includes nuclei (arrows).

To investigate the relevance of nuclear HIGD1A in vivo further, we examined infarcted mouse hearts generated utilizing a total occlusion model [50]. As an internal control for presence of ischemia we stained for AIF, which is known to translocate to the nucleus during severe ischemia [27]. As shown in Fig. 4, peri-infarct areas demonstrated diffused and nuclear AIF, whereas sites distal to the infarct demonstrated distinctly extranuclear AIF localization. Similar to these results, myocardial tissue surrounding the necrotic core of infarcted hearts demonstrated robust nuclear HIGD1A staining, whereas non-infarcted distal regions showed extranuclear HIGD1A localization. These *in vivo* results, together with the results in Fig. 3, suggest that nuclear localization of HIGD1A might be a widespread phenomenon during severe stress, and could potentially serve as a biomarker during these conditions.

### HIGD1A Localizes to the Nucleus in Peri-necrotic Tumor Regions in Cancer Xenograft*s in vivo*

**Figure 5:**
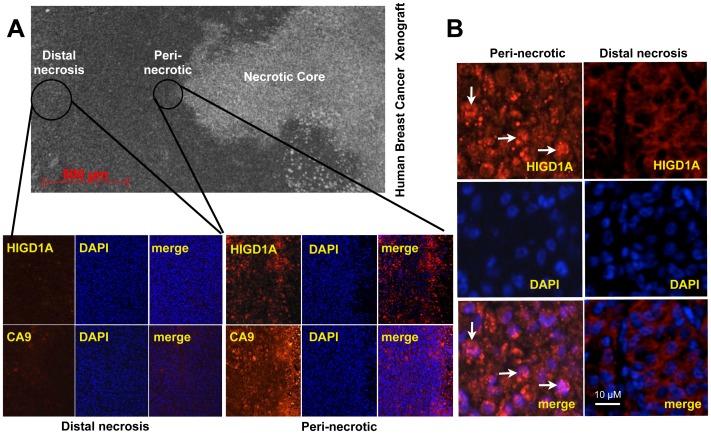
Nuclear localization of HIGD1A in mouse models of human breast cancer xenografts. (A) Top panel is a representative H&E stained slide of a human breast cancer xonograft indicating the perinecrotic region surrounding the necrotic core, as well as areas distal to the necrosis. Immunofluorescence microscopy analysis indicated that HIGD1A and the hypoxia marker CA9 were only minimally expressed distal to the region of necrosis, whereas both were highly expressed in the peri-necrotic region. (B) Perinecrotic regions contained predominantly nuclear (white arrows) localized HIGD1A, whereas areas distal to tumor necrotic regions had predominantly extranuclear HIGD1A. (B) Immunofluorescence microscopy of human gliobastoma xenografts demonstrating predominantly extranuclear HIGD1A before administration of Bevacizumab (pre-Bevacizumab), whereas after administration of Bevacizumab (post-Bevacizumab), HIGD1A becomes predominantly nuclear as indicated by white arrows.

Due to their rapid growth rates and defective vascularity, solid tumors are heterogeneous with respect to tissue oxygen and nutrient delivery. We examined HIGD1A expression in the human triple negative invasive breast cancer MDA-MB 231 xenografts that have previously been characterized and shown to contain anoxic perinecrotic regions [51]. As indicated by H&E staining in Fig. 5A, tumors contained necrotic regions. Peri-necrotic regions, which are known to be severely hypoxic, stained positive for the endogenous hypoxia marker CA9 as indicated by immunofluorescent microscopy. These same areas also stained strongly for HIGD1A. Regions distal to tumor necrotic areas stained weakly for CA9 and HIGD1A. As shown in Fig. 5B, perinecrotic tumor areas demonstrated nuclear HIGD1A localization, whereas distal regions to necrotic cores contained predominantly extranuclear HIGD1A.

### HIGD1A Localizes to the Nucleus in Human Glioblastomas after Antiangiogenesis Treatment

**Figure 6:**
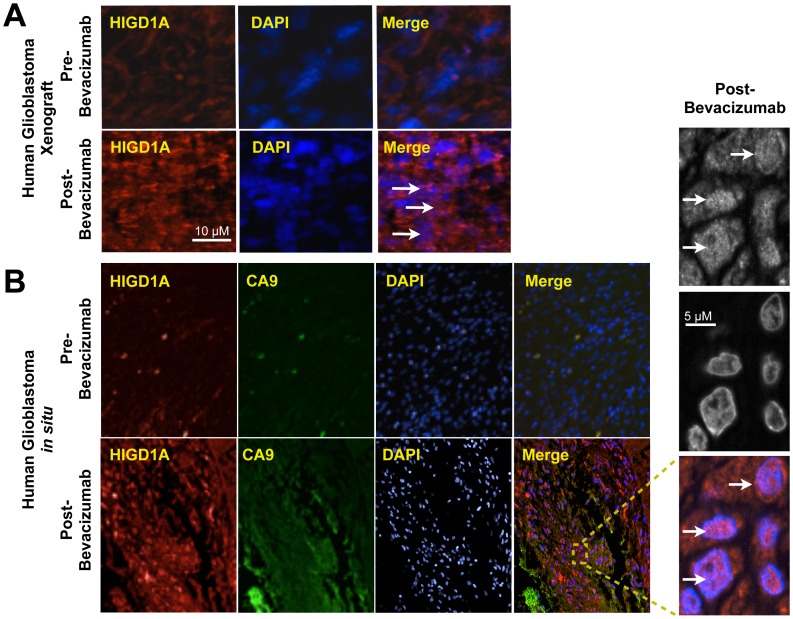
Nuclear localization of HIGD1A in response to Bevacizumab in human glioblastoma xenografts as well as glioblastoma patient biopsies. (A) Immunofluorescence microscopy of human glioblastoma xenografts showing HIGD1A expression and localization before (pre) and after (post) Bevacizumab treatment. White arrows indicate nuclear HIGD1A. (B) Immunofluorescence microscopy of paired human patient gliobastoma biopsies showing CA9 (hypoxia marker) and HIGD1A expression and localization before (pre) and after (post) treatment with the anti-angiogenic agent, Bevacizumab (Avastin). As indicated, HIGD1A was induced and predominantly nuclear in human glioblastoma samples after administration of Bevacizumab to patients. Lower levels of HIGD1A was expressed before treatment. As indicated in the inset HIGD1A localization to the nucleus is pronounced in glioblastoma after treatment with Bevacizumab (white arrows).

Anti-angiogenesis is currently being used in cancer therapy to disrupt tumor vascularization, which can result in cancer cell death due to induction of anoxia and severe ischemia. To assess the relevance of nuclear HIGD1A location in antiangiogenesis therapy, we first examined HIGD1A expression in glioblastoma xenografts before and after administration of Bevacizumab (Avastin). As indicated in Fig. 6A, before administration of Bevacizumab, HIGD1A was primarily extranuclear. However, after Bevacizumab treatment, HIGD1A also localized to the nucleus in these xenografts.

We further investigated the *in vivo* relevance of our xenograft observations in a human therapeutic setting that triggers significant tumor anoxia, ischemia, and hence, glucose starvation. Adaptive mechanisms that allow tumor cell survival following anti-angiogenesis treatments can compromise their therapeutic efficacy, highlighting the importance of understanding these survival pathways [52], [53] [54]. Therefore, to determine if HIGD1A was similarly induced in human glioblastomas following anti-angiogenesis treatment *in vivo*, we examined HIGD1A expression in human glioblastoma biopsies obtained before and after administration of Bevacizumab (Avastin) to patients. As shown in Fig. 6B, prior to the administration of Bevacizumab, both the hypoxia marker CA9 and HIGD1A levels were low. Localization of HIGD1A was primarily non-nuclear. However, after administration of Bevacizumab, hypoxic areas where created as indicted by increased CA9 staining. Under these conditions, HIGD1A expression was significantly increased, and was localized primarily to the nucleus, correlating with severe metabolic stress.

## Discussion

In this study, we have demonstrated the stress dependent nuclear localization of the HIF-1 target mitochondrial protein HIGD1A *in vitro* and *in vivo*. While physiological hypoxia promotes mitochondrial HIGD1A expression in a HIF-1-dependent manner, we found that severe metabolic stressors such as ischemia or DNA-damaging agents such as etoposide trigger nuclear accumulation of HIGD1A. Several mitochondrial factors such as AIF [55] and GAPDH [34] also become nuclear during conditions of severe stress, and the nuclear function of these factors modulates cell death pathways. While HIF-1 is generally considered to be an adaptive factor promoting cell survival during hypoxia, it can also promote cell death pathways via its target genes. BNIP3 is a mitochondrial factor [56], [57], and its expression is regulated by HIF-1 [58]. BNIP3 has primarily been described as a death factor, promoting apoptosis or autophagy [59], [60], although protective roles have also been described, depending on its subcellular localization. For example, in glioblastomas, BNIP3 has recently been localized to the nucleus [35], where it binds to the *Aif* gene promoter and represses its expression, thereby inhibiting AIF-mediated cell death [29], [30]. Further complicating the picture, AIF appears to also have dual nuclear roles. AIF can translocate from mitochondria to the nucleus and either induce apoptosis [46] or autophagy [61], which can promote cell death or survival, respectively. Similar to AIF and BNIP3, GAPDH is a mobile factor within the cell. Nuclear GAPDH can participate in cell death/dysfunction [31], [34], but can also have roles in cell survival via activation of DNA repair mechanisms, maintenance and protection of telomeric DNA from rapid degradation, and regulation of the redox state of a number of transcriptional regulators [28], [33], [62], [63]. The Hsp90-binding immunophilin FKBP51 is another mitochondrial protein that similarly becomes nuclear during stress, which then protects against oxidative stress [64]. Like AIF, GAPDH or FKP51, HIGD1A might also have novel nuclear roles that could fine tune cellular fates during conditions of severe stress.

Nuclear localization of mitochondrial proteins such as AIF is regulated in part by BAX and BAK mediated modulation of the mitochondrial outer membrane permeability [48], [65]. Our results suggest that nuclear localization of HIGD1A is similarly regulated by the presence of BAX and BAK. While AIF and GAPDH are believed to translocate directly from mitochondria to the nucleus, the localization of HIGD1A to the inner mitochondrial membrane makes this mechanism less likely. We surmise that a separate cytosolic pool of HIGD1A translocates to the nucleus during severe stress. Our biochemical fractionation experiments support this hypothesis, as mitochondrial HIGD1A levels did not decrease during apoptosis induction in MEFs (Fig. 2Ci).

Similar to our *in vitro* results, we also demonstrate nuclear-localization of HIGD1A during severe stress *in vivo*. Specifically, we show that in the setting of ischemic heart disease, hypoxic-ischemic encephalopathy and cancer, nuclear localization of HIGD1A correlates with severity of stress. Other HIF-1 targets such as Carbonic anhydrase 9 are endogenous markers of hypoxia and are upregulated in tumors after anti-angiogenesis treatment, and enable cell survival [52], [53]. Whether nuclear HIGD1A also promotes increased cell survival in these settings remains to be elucidated. Our results suggest, however, that it may potentially be a useful biomarker of pathological hypoxic/ischemic states *in vivo*.

## Methods

### Ethics Statement

Neonatal brain tissue was collected with written informed consent in accordance with guidelines established by the University of California San Francisco Committee on Human Research (Institutional Review Board IRB# H11170-19113-07). The CHR reviews research involving human subjects to ensure the ethical and equitable treatment of those subjects. Human tissue was obtained from autopsied material at the University of California San Francisco Medical Center following the general guidelines posted on http://www.research.ucsf.edu/chr/Guide/chrHumanBioSpec.asp#Research3.

Information about bevacizumab-resistant cases was obtained as part of a study approved by the UCSF Committee on Human Research (CHR). The CHR reviews research involving human subjects to ensure the ethical and equitable treatment of those subjects. Tissue from these cases was acquired from the UCSF Brain Tumor Research Center (BTRC), which obtains tissue after obtaining written informed consent from patients, a consent which allows the BTRC to distribute tissue to UCSF investigators. Human brain tumor tissue was obtained at the University of California San Francisco Medical Center following the general guidelines posted on http://www.research.ucsf.edu/chr/Guide/chrHumanBioSpec.asp#Research3.

### Cell Culture Conditions and Chemicals

Mouse embryonic fibroblasts (MEFs) were cultured in RPMI-1640 (Lonza), 10%FBS, 2.5 µg/ml Fungizone, 100 µg/ml Penicillin/Streptomycin, and 110 µg/ml Sodium Pyruvate. MEFs have been described in [66]. *Bax/Bak* ^−/−^ MEFs were from obtained from N. Chandel. Fungizone, Penicillin/Streptomycin, and Sodium Pyruvate were from the UCSF Cell Culture Facility. Glucose starvation was achieved by culturing cells in MEF media utilizing glucose free RPMI 1640 (Lonza). Cells were harvested via trypsinization using 0.25% trypsin with EDTA also sourced from the UCSF Cell Culture Facility. Cells were incubated in a tissue culture incubator at 5% CO_2_ and 21% O_2_ while hypoxic experiments were performed for 20 hours at 2% or 1% O_2_ with 5% CO_2_ using a HERA-cell 240 (Thermo Electron Corp), or an XVivo hypoxia workstation(Biospherix). Oxygen level was monitored with inbuilt oxygen sensors or by using an Analox oxygen indicator (Analox). Cells were incubated in RPMI for 24 hours and 40 µM etoposide (Sigma) was added to the cells for indicated time points.

### Cellular Fractionation Extracts

Cells were seeded overnight to achieve a density of approximately 80%, and then treated with etoposide for 12 hours. Fractions were made by using the MS861 Cell Fractionation Kit per manufacturer’s instructions (MitoSciences), with slight modification, where the final nuclear pellet was completely lysed with the use of a sonicator.

### Immunoblot Analysis

For SDS-PAGE, whole cell lysates were prepared in a cold room (4°C). Lysates were prepared by using Urea lysis buffer (8 M urea, 10% glycerol, 5 mM DTT, 10 mM Tris-HCl pH 6.8, 1% SDS, and 1x Proteinase Inhibitor Cocktail (Roche Diagnostics)) by adding lysis buffer directly to cells that were washed with ice cold PBS. Lysates were sonicated to ensure complete lysis. Protein levels were quantified using the bicinchoninic acid (BCA) Protein Assay (Thermo Scientific Pierce). Whole cell lysates (20–100 µg protein per lane) were subjected to gel electrophoresis on 7.5%, 12% or 15% Gold Pre Cast PAGEr gels (Lonza) and blotted onto Immobilon-FL membranes (Millipore) using semi-dry transfer (Bio-Rad). Membranes were blocked in blocking buffer from LI-COR Biosciences and probed with primary antibodies in LI-COR blocking buffer. Primary antibodies were: murine HIGD1A (Proteintech Group), human HIGD1A (Santa Cruz), GFP (Invitrogen), GAPDH (Novus), Complex IV (Mitosciences), Histone H3 (Abcam), AIF (Cell Signaling), BNIP3 Cell Signaling). For secondary antibodies, IRDye 800CW goat anti-rabbit and IRDye 680 goat anti-mouse secondary antibodies (LI-COR Biosciences) were used in LI-COR blocking buffer supplemented with SDS and Tween-20 according to the manufacturer’s protocol. Proteins were visualized in conjunction with the LI-COR Odyssey Imaging System for signal detection.

### Plasmid Constructs

The murine HIGD1A cDNA was obtained from Origene (Image accession number 5148784) and used for all subsequent expression constructs. HIGD1A fusion proteins were generated through overlap extension PCR, cloned into the ENTRD-TOPO vector (Invitrogen) and confirmed by DNA sequencing. For HIGD1A-GFP, monomeric EGFP (Karel Svoboda, Addgene Plasmid 18696) was fused to the C-terminus of HIGD1A via a 25 amino acid tetrameric helical linker (HL4) [67]. For expression in cell culture, a derivative of the Piggybac transposon system from [68] was employed allowing high efficiency expression. The parental plasmid EBXN containing the minimal Piggybac 5′ and 3′ inverted terminal repeats as well as a CMV enhancer chicken Beta-actin promoter expression cassette was modified to include the SV40 promoter Blasticidin cassette allowing for eukaryotic selection in cell culture. The plasmid was further modified to include the Invitrogen Gateway Rfa cassette allowing for phiC31 mediated recombination. HIG1DA-GFP was cloned into PBX2.2.

Transfection was performed with Lipofectamine LTX and PLUS reagent (Invitrogen). A 2∶1 molar ratio of Piggybac transposase helper plasmid was combined with the transposon expression construct to mediate integration and high level expression. Selection with Blasticidin 10 µg/ml was performed to select for stable integrants. Selected cells for expression of GFP or HIGD1A-GFP were frozen and thawed when needed for experiments.

### Immunohistochemistry and Microscopy

Staining of cells: Cells grown on microscope cover glass were fixed in ice cold methanol for 15 minutes at −20°C, after which they were washed with PBS. Blocking was performed in BSA/PBS/Tween20 for 1 hour, after which primary antibody (1∶300 dilution) in BSA/PBS without Tween20 was added to the cells for 1 hour. Cells were then washed with PBS, and then secondary AlexaFluor antibody was added in BSA/PBS/Tween20 solution for 1 hr. Cells were washed with PBS and mounted with Vectashield mounting solution containing DAPI.

Staining of tissue samples: Neonatal HIE brain cryosections were cut at 20 µm. Paraffin embedded heart and MDA-MB 231 tumor sections were cut at 2 and 5 µm respectively. Glioblastoma paraffin embedded sections were cut at 16 µm. Paraffin embedded sections were heated to 95°C for antigen retrieval in 0.01 M Citrate buffer, pH 6.0, and blocked with 10% normal goat serum in 1.5% Triton X-100/PBS for 1 hour at room temperature. Sections were incubated overnight at 4°C in primary antibody in 10% goat serum and 0.5% Triton X-100/PBS. AlexaFluor Secondary fluorescent antibodies (Invitrogen) were used for immunofluorescent detection.

Cells were visualized in the UCSF Biological Imaging Development Center utilizing a spinning disk confocal microscope (Zeiss Axiovert microscope, Yokogawa CSU10 confocal scanner unit), or with a Zeiss Imager Z.2 fluorescence microscope (Karl Zeiss) equipped with an Apotome and axiovision software for optical sectioning.

### Tissue Samples

#### Patient Glioblastoma

Additional information on Glioblastoma biopsies and bevacizumab treatments can be obtained in detail from [52], [69]. Briefly, tumors from patients at recurrence but before bevacizumab treatment were as pre-Avastin/bevacizumab section. Avastin was administered at 10 mg/kg and tumors were followed with MRI. Once the tumor became resistant (no longer shrinking, more infiltrating) tumors were surgically removed and harvested with the resulting tissue considered post-bevacizumab. For glioblastoma xenografts, 500 K U87 MG tumor cells were injected subcutaneously into 10 athymic nude mice and 5 mice were treated with Avastin/Bevacizumab (10 mg/Kg) twice a week, and the other 5 mice with Human IgG (10 mg/Kg). Mice were sacrificed when tumors had reached a size of ≥2 cm per IACUC protocol. Tumors were fixed in 1% PFA overnight then allowed to sink completely in 50 mL of 30% Sucrose. They were then frozen in blocks of OTC and sectioned at 20 µm.

#### Murine myocardial infarction

Myocardial infarction utilizing a total occlusion model was induced in mice as described in [50].

#### MDA-MB 231 breast Cancer Xenografts

Xenografts were generated at Stanford University, approved by Stanford’s Institutional Animal Care and Use Committee and in accordance with all Administrative Panel on Laboratory Animal Care (APLAC) regulations at Stanford University and were in compliance with the National Institutes of Health Guide for Care and Use of Animals, and have been described in [51]. Briefly, NOD/SCID (non-obese diabetic – severe combined immunodeficiency) female mice had MDA-MB-231 breast cancer cells in 100 ml phosphate buffered saline (PBS; pH 7.4) plus 100 ml of matrigel (BD Biosciences, San Jose, CA, USA) injected into their left second mammary fat pads. At 55 days after injection, when the average tumor volume was 1.5 cm mice were euthanized for and tumors excised and embedded in paraffin.

#### Neonatal hypoxic brain tissue

Following autopsy, brains were immersed in phosphate buffered saline with 4% paraformaldehyde for three days. On day 3, the brain was cut in the coronal plane at the level of the Mamillary Body and immersed in fresh 4% paraformaldehyde/PBS for an additional three days. Post fixation, all tissue samples were equilibrated in PBS with 30% sucrose for at least 2 days. Following sucrose equilibration, tissue was placed into molds and embedded with OCT for 30 to 60 minutes at room temperature or 4°C followed by freezing in dry ice-chilled ethanol or methyl butane. The diagnosis of hypoxic ischemic encephalopathy (HIE) requires clinical and pathological correlations. With respect to the pathological features, all HIE cases in this study showed consistent evidence of diffuse white matter injury, including astrogliosis and macrophage infiltration. These findings were confirmed by the increase in the number and the staining intensity of GFAP- or CD68-positive cells, respectively (not shown). In addition, HIE cases also showed evidence of neuronal injury, including the presence of ischemic neurons and variable degrees of neuronal loss, in cerebral cortex, hippocampus and basal ganglia (not shown).

### Immunoprecipitation Assays

Adherent cells were washed twice by addition of ice cold PBS to the monolayer and disposal of the supernatant. 1 ml of freshly made ice cold lysis/wash buffer (50 mM Tris-HCl, 150 mM NaCl pH 7.5, 1% Nonidet P40 0.5% sodium deoxycholatex supplemented with 1 complete tablet from Roche) was added to the washed cell monolayers to achieve a concentration of 10^6^–10^7^ cells/ml. Cells were scraped into an eppendorf, and sonicated on ice with 5 pulses each for 8 seconds long. Lysate was spun down at 13000 rpm for 5 minutes. Supernatant (except 200 µl) was put onto a new tube. The un-lysed pellet was resuspended into the 200 µl remaining lysate, and sonicated again, the tube centrifuged at 13000 rpm for 5 minutes and the new lysate added to the original lysate. This was repeated three times until complete lysis was achieved. 50 µl of this lysate was kept aside as input. To reduce background a preclearing step was performed overnight. 50 µl of the homogeneous protein G- agarose (Roche) suspension, equilibrated in the lysis buffer was added to the 1 ml lysate at 2–8°C on a rotating platform overnight. Beads were then pelleted by centrifugation at 2000×g for 2 minutes at 4°C. Supernatant was transferred to a new tube. 50 µl of Chromotek-GFP-Trap bead (Allele Biotechnology), a GFP-binding protein based on a single domain antibody derived from Lama alpaca, was equilibrated in the wash/lysis buffer, centrifuged for 2 minutes at 2000×g, and supernatant discarded. The cell lysate was added to these beads and rotated (gentle end-over-end mixing) for 1 hour at 4°C. The lysate/bead complex was then centrifuged for 2 minutes at 2000×g. Pellet was washed 4x by resuspending in 1 ml lysis/wash buffer. A final wash was performed once for 30 minutes by end-over-end mixing. Beads were then resuspended in 90 µl of 2x SDS pro-track sample buffer (Lonza), boiled for 10 minutes at 95°C. Beads were collected by centrifugation at 2700×g for 2 minutes at 4°C and SDS-PAGE performed with the supernatant.
